# Ontology-Based Healthcare Named Entity Recognition from Twitter Messages Using a Recurrent Neural Network Approach

**DOI:** 10.3390/ijerph16193628

**Published:** 2019-09-27

**Authors:** Erdenebileg Batbaatar, Keun Ho Ryu

**Affiliations:** 1 College of Electrical and Computer Engineering, Chungbuk National University, Cheongju 28644, Korea; 2 Faculty of Information Technology, Ton Duc Thang University, Ho Chi Minh City 700000, Vietnam; 3 Database and Bioinformatics Laboratory, Department of Computer Science, College of Electrical and Computer Engineering, Chungbuk National University, Cheongju 28644, Korea

**Keywords:** named entity recognition, healthcare, deep learning, recurrent neural network, word embedding, ontology, unified medical language system, conditional random field, Twitter

## Abstract

Named Entity Recognition (NER) in the healthcare domain involves identifying and categorizing disease, drugs, and symptoms for biosurveillance, extracting their related properties and activities, and identifying adverse drug events appearing in texts. These tasks are important challenges in healthcare. Analyzing user messages in social media networks such as Twitter can provide opportunities to detect and manage public health events. Twitter provides a broad range of short messages that contain interesting information for information extraction. In this paper, we present a Health-Related Named Entity Recognition (HNER) task using healthcare-domain ontology that can recognize health-related entities from large numbers of user messages from Twitter. For this task, we employ a deep learning architecture which is based on a recurrent neural network (RNN) with little feature engineering. To achieve our goal, we collected a large number of Twitter messages containing health-related information, and detected biomedical entities from the Unified Medical Language System (UMLS). A bidirectional long short-term memory (BiLSTM) model learned rich context information, and a convolutional neural network (CNN) was used to produce character-level features. The conditional random field (CRF) model predicted a sequence of labels that corresponded to a sequence of inputs, and the Viterbi algorithm was used to detect health-related entities from Twitter messages. We provide comprehensive results giving valuable insights for identifying medical entities in Twitter for various applications. The BiLSTM-CRF model achieved a precision of 93.99%, recall of 73.31%, and F1-score of 81.77% for disease or syndrome HNER; a precision of 90.83%, recall of 81.98%, and F1-score of 87.52% for sign or symptom HNER; and a precision of 94.85%, recall of 73.47%, and F1-score of 84.51% for pharmacologic substance named entities. The ontology-based manual annotation results show that it is possible to perform high-quality annotation despite the complexity of medical terminology and the lack of context in tweets.

## 1. Introduction

An overwhelming amount of health-related knowledge has been recorded in social media sites such as Twitter, with the number of tweets posted each year increasing exponentially [1,2,3]. Twitter is the most comprehensive social media site collecting and providing public health information: 500 million tweets are sent each day—5000 every second. Although a large amount of information is thought to be reliable for monitoring and analyzing health-related information, the lack of methodological transparency for data extraction, processing, and analysis has led to inaccurate predictions in detecting disease outbreaks, adverse drug events, etc. As a result, health-related text mining and information extraction are active challenges for the development of useful public health applications for researchers [4,5,6]. One essential part of developing such an information extraction system is the NER process, which defines the boundaries between common words in terminology in a particular text, and assigns the terminology to specific categories based on domain knowledge [7,8,9].

NER, also known as entity extraction, classifies named entities that are present in a text into pre-defined categories like “location”, “time”, “person”, “organization”, “money”, “percent”, and “date”, etc. [10]. An example is as follows: (ORG U.N.) official (PER Ekeus) heads for (LOC Baghdad) [11]. This sentence contains three named entities: Ekeus is a person, the U.N. is an organization, and Baghdad is a location.

In the traditional NER method based on machine learning, part-of-speech (POS) information is considered as a key feature of entity recognition [10,11,12,13]. In 2016, Lample et al. [7] presented a neural architecture based on long short-term memory (LSTM) that uses no language-specific resources and hand-engineered features. They compared the LSTM and conditional random fields (LSTM-CRF) model and stack LSTM (S-LSTM) model with various NER tasks. The state-of-the-art NER systems for English produce near-human performance with an F1 score of over 90%. For example, the best system entering Seventh Message Understanding Conference (MUC-7) in [14] scored 93.39% for the F-measure, while human annotators scored 97.60% and 96.95%. However, the performances in the healthcare, biomedical, chemical, and clinical domains are not as good as the performances in the English domain. They are restricted by problems such as the number of new terms being created on a regular basis, the lack of standardization of technical terms between authors, and by the fact that technical terms (for example, disease, drugs, and symptoms) often have multiple names [15]. Consequently, state-of-the-art NER software (e.g., Stanford NER) is less effective on Twitter NER tasks [9].

Public health research requires the knowledge of disease, drugs, and symptoms. Researchers focus on exploring population health, well-being, disability, and the determining factors for these statuses, be they biological, behavioral, social, or environmental. Moreover, researchers develop and assess interventions aiming to improve population health, prevent disease, compensate for disabilities, and provide innovations in terms of the organization of health, social, and medical services [16]. The Internet has revolutionized efficient health-related communication and epidemic intelligence [17]. People are increasingly using the Internet and social media channels. In the modern world of social media dominance, microblogs like Twitter are probably the best source of up-to-date information. Twitter provides a huge amount of microblogs, including health information that are completely public and pullable.

The purpose of the research reported in this paper was to predict health-related named entities such as diseases, symptoms, and pharmacologic substances from noisy Twitter messages that are essential for discovering public health information and developing real-time prediction systems with respect to disease outbreak prediction and drug interactions. To achieve this goal, we employed a deep learning approach obtaining the pre-trained word embedding which can be used successfully for any text mining tasks. We collected a large number of Twitter data, and then cleaned and preprocessed them to produce an experimental dataset. We automatically annotated the dataset using the UMLS Metathesaurus [18] with three types of entities (diseases, symptoms, and pharmacologic substance). Our deep learning architecture follows the window approach in [19]. The method we put forward has a number of desirable advantages:

1. We achieved a precision of 93.99%, recall of 73.31%, and F1-score of 81.77% for disease or syndrome HNER; a precision of 90.83%, recall of 81.98%, and F1-score of 87.52% for sign or symptom HNER; and a precision of 94.85%, recall of 73.47%, and F1-score of 84.51% for pharmacologic substance named entities using the BiLSTM-CRF model.
2. The architecture uses little hand-engineered features using POS tagging. Therefore, it has a great capability for improving state-of-the-art performances.
3. We presented a large number of tweets on the HNER task using domain-specific UMLS ontology, including three health-related entity types (diseases, symptoms, and pharmacologic substance).
4. The health-related domain (including disease, syndrome, sign, symptom, and pharmacologic substance) was particularly well applied because the BiLSTM-CRF could extract health-related entities and identify the relationship between them from Twitter messages.

The remainder of the paper is organized as follows: Section 2 introduces the theoretical foundation of this paper and related works. Section 3 focuses on the detailed description of the experimental dataset, health-related named entity recognition tasks, and how the deep learning model is trained. In Section 4, the experimental analysis and the related results are provided. Finally, Section 5 provides a discussion about the experimental analysis and address our conclusion.

## 2. Background

### 2.1. Research Framework

**Figure 1:**
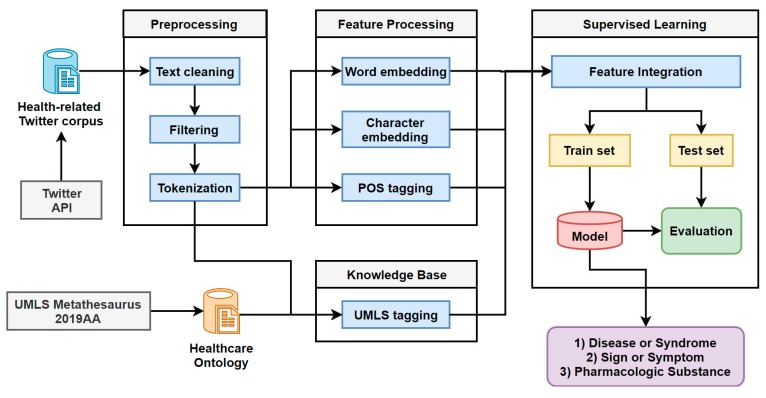
Overflow of HNER. API: application programming interface.

In this paper, we present an HNER task using healthcare-domain ontology. Figure 1 shows the overflow of HNER task. For the input of the HNER task, we created a healthcare Twitter corpus which was collected from Twitter with the search term “healthcare” between 12 July 2018 and 12 July 2019. Firstly, we used the basic preprocessing techniques such as text cleaning including removing hashtags and Uniform Resource Locators (URLs), removing punctuation, and eliminating multiple white spaces and text normalization. We used text filtering to avoid a large number of false positives. Only tweets with the three named entities (“disease”, “symptom”, and “pharmacologic substance”) were kept and tweets with common non-medical words such as “fit”, “water”, “others”, “may”, and “said” etc., were removed. Then we used tokenization for the word-level sequence. Secondly, we produced word-level and character-level features. For word-level features, we used pre-trained word embeddings and POS tagging methods, and the CNN was used to produce character-level features.

Additionally, for getting knowledge of healthcare domain ontology, we used UMLS tagging to create a label for a sequence of inputs. Finally, we integrated all the features and the combinations of the features for experiments. We split the experimental dataset into training and testing sets. LSTM-CRF and BiLSTM-CRF models were trained on the training set and evaluated on the testing set. In the scope of the HNER task, the trained models could recognize medical entities from Twitter data. For example, given the sequence of input tweet “Last week, President Donald Trump declared the opioid crisis a national public health emergency”, NER systems would only recognize the person (Donald Trump) and fail other health-related entities. For solving this, BiLSTM-CRF model can recognize the medical entity (opioid crisis) that is required in public health research.

### 2.2. Related Work

Information extraction is the process of extracting useful information such as the relationship between entities from unstructured or raw data [20]. This process of extraction of structure from noisy sources like microblogs (e.g., Twitter) is indeed challenging [21]. For instance, tweets are typically short. The number of characters in a particular tweet is restricted to 140 characters, and the contextual information is limited. Recently, various deep learning architectures have been applied to fields like computer vision, automatic speech recognition, natural language processing, and music/audio signal recognition, where they have been shown to produce state-of-the-art results on various tasks. In Natural Language Processing (NLP) tasks including tasks such as NER [10], POS tagging [22], Semantic Role Labeling [23], Dependency Parsing [24], Sentiment analysis [25], and Web Search, etc., this is particularly true [20,26]. In BioNLP [27,28,29] tasks, deep learning techniques have been studied successfully [30,31]. These advances in deep learning have inspired novel approaches for a better understanding of healthcare. Deep learning models have been demonstrated to provide a significant improvement in predictive modelling when resuming the properties and activities of disease, symptoms, and drug discovery [32,33,34].

Over the last few years, a number of deep learning architectures have been proposed in the biomedical and chemical NER field. There is a lack of deep learning methods for health-related NER tasks from social media sites like Twitter. Mainly the approaches cover the CNN [35,36,37], the recurrent neural network (RNN) [32,38,39], and the combination of the two architectures (CNN-RNN [40]). Nowadays, NER approaches struggle with generalization problems in specific fields. Convolutional neural network models generally capture local features that are hard to solve. That is why the combined CNN-RNN [40] model has been proposed for generalization. Recently, LSTM, a particular case of the RNN model, has been successfully developed in NLP and biomedical text mining tasks. LSTM with CRF [32,38] models have achieved the improved results in the biomedical named entity recognition task. Very recently, an advanced deep neural network type called BiLSTM has increasingly been employed in studies of biomedical NER, yielding state-of-the-art performance at the time of their publication [32,38,41,42,43]. Moreover, the attention-based BiLSTM-CRF model is proposed as well to capture similar entity attention at the document level [44]. One of the well-known deep learning-related methods is word embeddings.

Word embedding [45] is a function to map words to high-dimensional vectors. At present, a neural network is one of the most-used learning techniques for generating word embedding [46]. Word embedding helps to understand how different words are related based on the context. In healthcare, mapping of biomedical entities into a representation space is used to find a relationship between named entities in text corpora [47]. Since any deep architecture is based on word embedding, the use of word embedding in an unsupervised fashion on a large collection of text has become a key “secret sauce” for the success of many NLP systems using deep learning in recent years. The word embedding computed using neural networks explicitly capture many linguistic regularities and syntactic patterns.

Even though a number of methods for health-related NER from twitter messages for public health and HNER tasks have been presented, deep learning techniques have been insufficiently studied. There are some successful works applying NER analysis to Twitter [9,13,48]. A few works are concentrated on health-related entities including disease, drugs, and symptoms [49] and applied neural network architectures [50]. Ontology-based deep learning techniques also successfully applied to extract disease names from Twitter messages [51]. The recent works have mostly used a small number of a dataset. In this paper, we leveraged a large number of tweets and applied the BiLSTM-CRF model to the HNER task by taking advantage of deep learning on large training observations. Therefore, to encourage researchers to use deep learning for healthcare text mining, we designed a useful a large annotated dataset and prediction approach.

To best of our knowledge, the HNER task was most recently introduced by Jimeno-Yepes et al. [49], and they presented Micromed dataset. Later, Jimeno-Yepes and MacKinlay [50] applied LSTM-CRF model to the Micromed dataset. In this paper, we present a dataset that is larger than the Micromed, employing various RNN techniques and providing comprehensive results.

## 3. Materials and Methods

### 3.1. Dataset

We have obtained a large number of health-related twitter data through Twitter API [52] using the search term “healthcare” between 12 July 2018 and 12 July 2019. The dataset contains 1,403,393 health-related tweets.

For the HNER task, we only considered the three types of entities such as diseases, symptoms, and pharmacologic substances to match the particular entities we target for annotation. These types of entities are also annotated in Micromed dataset [49]. Table 1 shows the detail of each entity type. We found 189,517 tweets for “disease or syndrome”, containing 382,629 medical terms (7.25% of total words) and 9536 unique terms (3.74% of total unique words). There were 77,466 tweets found for “sign or symptom”, containing 99,367 medical terms (4.33% of total words) and 2043 unique terms (4.56% of total unique words). A total of 409,268 tweets were found for “pharmacologic substance”, containing 848,871 medical terms (7.51% of total words) and 8148 unique terms (1.80% of total unique words). Examples of tweets and corresponding medical terms are as shown below:

Example 1: “Cannabis (T121) Strains (T121) to beat stress (T184) after recommendations from Marijuana (T121) doctors in Los Angeles”.

Example 2: “Join VLAB on February 26th to learn more about the breakthroughs in diabetes (T047) like the artificial pancreas (T047)”.

Example 3: “Nightmare (T184), narcolepsy (T184) and sudden (T184) weakness (T184) turn Mary’s life upside down after swine flu (T047) vaccination”.

In the preprocessing step, we removed all URLs (starting with “http” and “https”), hashtags (starting with “#”), non-English characters, and punctuation. Then we converted all characters to lower case. Finally, we only selected the tweets containing at least five words. Not all tweets contained health-related entities. We filtered out tweets using a list of medical terms in UMLS. We only kept the tweets if it contained at least one entity from the medical entity types, and the others were removed.

Finally, we filtered 676,251 tweets with a total of 1,330,867 medical terms and 19,727 unique medical terms for our experiment. The tweets in the experimental dataset contain at least one health-related entity. The health-related entities in each entity type and frequency are shown in Table 2. To avoid a large number of false positives, we removed the following non-medical terms from each entity type:

- T047: condition, best, recruitment, disease, may, said, founder, increasing, west, evaluable, etc.
- T184: fit, weight, finding, catch, imbalance, medicine, others, walking, spots, mass, etc.
- T121: water, various, program, drugs, stop, tomorrow, orange, support, solution, speed, etc.

We joined the relevant Metathesaurus table (“MRCONSO.RRF” and “MRSTY.RRF”) to determine health-related named entities. We normalized all terms in tweets using the Jaccard similarity measure (>0.7):

- T047: diabet to diabeta (0.80), alzheime to alzheimer (0.86), obesit to obesity (0.80), etc.
- T184: strains to strain (0.80), grimaced to grimace (0.83), illnesss to illness (0.83), etc.
- T121: marijuan to marijuana (0.86), pharmaceutica to pharmaceutical (0.71), etc.

After all, preprocessing and filtering, we split the experimental dataset into training, testing, and validation subsets. Table 3 shows the distribution of tweets and the corresponding number of tweets, number of terms, and unique terms for each entity type.

### 3.2. Dataset Annotation Tool

For dataset annotation, we used QuickUMLS tool [53] to extract biomedical concepts from medical text. We use downloaded the latest version of UMLS (umls-2019AA-metathesaurus) and set the parameters as shown in Table 4.

### 3.3. Health-Related Named Entity Recognition

**Figure 2:**
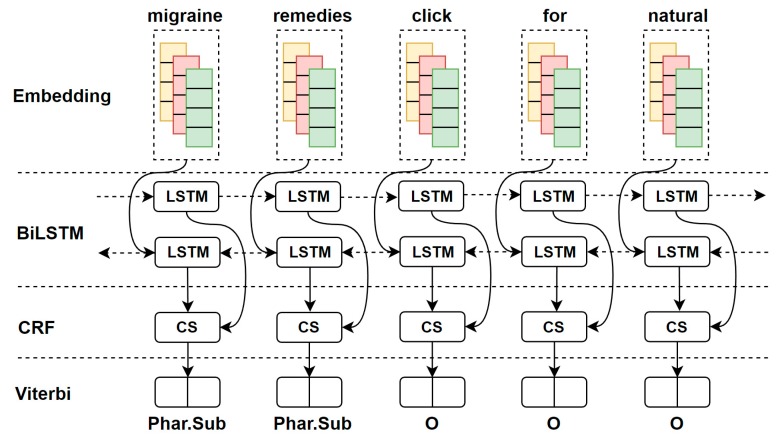
The BiLSTM-CRF model. CS: confidence score; BiLSTM: bidirectional long short-term memory; CRF: conditional random field.

In this section, we provide the problem definition in HNER, the details of BiLSTM-CRF model architecture and the process of the training. We apply the Pytorch library [54] to implement our model. Our main goal is to predict medical terms in given sentences or tweets. The overview of BiLSTM-CRF model is shown in Figure 2. BiLSTM-CRF model consists of four layers including the embedding, BiLSTM, CRF, and Viterbi layers. The embedding layer consists of the three sub representations such as word embedding features (yellow), character features (red), and additional word features (green). The medical and non-medical pre-trained word embeddings are used and compared for producing word embedding. CNN is used for producing character embedding, and POS tagging is used for producing additional word features. BiLSTM learns the contextual information from the concatenated word and character representations, and generates the word-level contextual representations that indicate the confidence score “CS” for each word. The CRF layer calculates tagging scores for each word input based on the contextual information. Finally, the Viterbi algorithm is used to find the tag sequence that maximizes the tagging scores. We explain the details of the presented model in the next sections and how it applies to the HNER task.

#### 3.3.1. Problem Definition

We consider named entity recognition as a combination of two problems: segmentation and sequence labelling, given

- an ordered set of N character sequences $X=\left(X_{1},\;X_{2},\;\ldots ,X_{N}\right)$, where $X_{i}=\left(c_{1}^{i},c_{2}^{i},\ldots ,c_{n}^{i}\right)$ is a character sequence;
- an ordered set of N annotations $Y=\left(Y_{1},\;Y_{2},\;\ldots ,Y_{N}\right)$, where $Y_{i}$ is a sequence $Y_{i}=\left(y_{1}^{i},y_{2}^{i},\ldots ,y_{n}^{i}\right)$ and $y_{j}^{i}$ is a tuple of two boolean labels ($s_{j}^{i},e_{j}^{i}$) showing whether the corresponding character is the beginning of a chemical entity and/or part of one, respectively.

Our task is to create a predictor $P:X\to \hat{Y}$, where $\hat{Y}$ is a set of inferred annotations similar to Y. We also use a tokenizer: $X\to \tilde{X}$, where $\tilde{X}$ is an ordered sequence of character subsequences (tokens), thus slightly redefining the objective function to target per-token annotations. Provided that the tokenizer is fine enough to avoid tokens with overlapping annotations, this redefined problem is equivalent to the original one.

#### 3.3.2. Feature Representation

In the first phase of the prediction model, named as embedding, we represent each token by word embedding (1), character embedding (2), and POS tagging (3).

**Word Embedding (word):** We used both non-biomedical and biomedical pre-trained word embedding and analyzed the effect of word embedding for the HNER task. In this paper, we used non-medical word embedding with GloVe [55] and Word2Vec [56]. We also used medical word embedding as found in Pyyssalo et al. [57], Chiu et al. [47], Chen et al. [58], and Aueb et al. [59]. Our experimental results show the comparison of these word embedding on the healthcare NER task from Twitter. The details are explained in Appendix A and the statistics of word embedding are described in Table A1 and Table A2.

**Figure A1:**
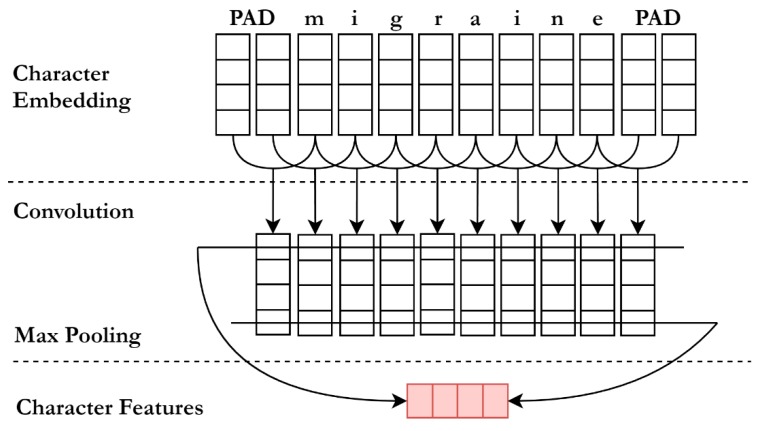
CNN for extracting character-level features.

**Character Embedding (char):** Character-level word embedding is useful, especially when rich rare words and out-of-vocabulary words are exploited and word embedding is poorly trained. It is common in the biomedical and chemical domain. Word-level approaches fall short when applied to Twitter data, where many infrequent or misspelled words occur within very short documents. We considered character-level word embedding in this paper. The details are explained in Appendix B and. Also, Table A3 shows the character set used in this paper and Figure A1 shows the CNN for extracting character-level features.

**Additional word feature (POS):** Most state-of-the-art NER systems [39,60] use additional features such as POS tagging [61] as a form of external knowledge. We also used POS tagging as an additional word feature in this paper. POS tags are useful for building parse trees, which are used in building NERs and extracting relations between words. Table 5 shows an example of how POS features are applied.

#### 3.3.3. Feature Learning

**Figure A2:**
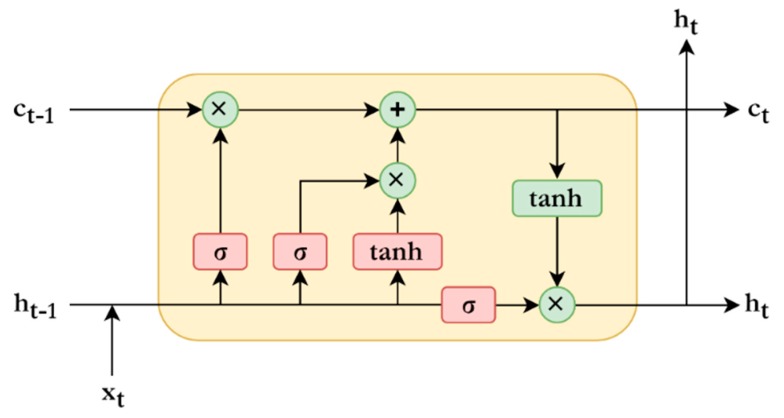
LSTM memory cell.

After concatenating the different feature representations, we employed the BiLSTM layer to learn sequential structure of words in tweets. LSTM and BiLSTM have commonly used RNN techniques in NLP tasks. In comparison with a single-direction LSTM, a BiLSTM can use the information from both sides to learn the input features. The details are explained in Appendix C and Figure A2 shows the LSTM memory cell in detail.

#### 3.3.4. Prediction

After learning the input features, the famous CRF layer is employed. BiLSTM-CRF is the combination between BiLSTM and CRF, a string algorithm for sequence labelling tasks which is very effective. In a BiLSTM model, the tagging decision at the output layer is made independently using a softmax activation function. That means the final tagging decision of a token does not depend on the tagging decision of others. Therefore, adding a CRF layer into a BiLSTM model equips the model with the ability to learn the best sequence of tags that maximizes the log probability of the output tag sequence. BiLSTM-CRF is very successful for NER tasks. They produce the state-of-the-art results on several NER benchmark data sets without using any features. The details are explained in Appendix D and Appendix E.

### 3.4. Network Training

In this section, we provide the detail process of our neural network training. We apply the Pytorch library to implement the LSTM-CRF and BiLSTM-CRF models.

We train our network architecture with the back-propagation algorithm [62] to update the parameters for each training example using the work of Adam [63] with Nesterov momentum [64]. In each epoch, we divide all the training data into batches, then process one batch at a time. The batch size decides the number of sentences. In each batch, we firstly get the output scores from the BiLSTM for all labels. Then we put the output scores into CRF layer, and we can get the gradient of outputs and the state transition matrix. From this, we can backpropagate the error from output to input, which contains the backward propagation for bi-directional states of LSTM. Finally, we update all the parameters.

Dropout [65] can mitigate the overfitting problem. We apply dropout on the weight vectors directly to mask the final embedding layer before the combinational embedding feed into the bi-directional LSTM. We fix the dropout rate at 0.5 as usual and achieve good performance on our model. We also use the early stopping strategy with patience 20 to avoid overfitting the early stopping monitored weighted F1-scores on validation sets.

### 3.5. Hyparameter Settings

Our hyper-parameters are shown in Table 6. We used three-layer convolution and set the output of the convolution layer to 50 for extracting character features from each word. We also used two-layer LSTM and set the state size of LSTM to 250. For stopping condition, we used an early stopping strategy, and maximum iteration has been set at 100. The batch size is 100, the dropout layer is 0.5, and the initial learning rate is 0.001.

The experimental hardware platform was the Intel Xeon E3 (32G memory, GTX 1080 Ti). The experimental software platform was the Ubuntu 17.10 operating system and the development environment was the Python 3.5 programming language. The Pytorch library and the Scikit-learn library of Python were used to build the healthcare NER recognition model and comparative experiments.

### 3.6. Evaluation Metrics

For evaluating our model, an exact matching criterion was used to examine three different result types. False-negative (FN) and false-positives (FP) are incorrect negative and positive predictions, respectively. True-positive (TP) results corresponded to correct positive predictions, which are actual correct predictions. The evaluation is based on the performance measures precision (P), recall (R), and F-score (F). Recall denotes the percentage of correctly labelled positive results overall positive cases and is calculated as:

(1) P=TP/(TP+FP)

(2) R=TP/(TP+FN)

(3) F=(2×P×R)/(P+R)

## 4. Results and Discussion

In this paper, we employed the BiLSTM-CRF model with different combinations of word features (word embedding, character embedding, and POS tagging) for the divided dataset. The BilSTM-CRF model is compared with LSTM-CRF model presented by Jimeno-Yepes and MacKinlay [50] for the most similar task. To best of our knowledge, there are no other published works which use Twitter data for the health-related NER task. They used LSTM-CRF model with a pre-trained word-embedding and outperformed CRF model on the Micromed dataset. We present a dataset similar to Micromed, but our dataset is larger. Larger datasets support deep learning methods to improve the complexity of the problem and of the learning algorithm. The comparative performance evaluation result is shown in Table 7. The disease or syndrome HNER performance of BiLSTM-CRF (word + char + POS) has a precision of 93.99%, recall of 73.31%, and F1 of 81.77% when evaluating on the presented dataset. BiLSTM-CRF (word + char) has a precision of 94.53%, and LSTM-CRF (word + char + POS) has an F1 of 82.08%. The sign or symptom HNER performance of BiLSTM-CRF (word + char + POS) has a precision of 90.83%, recall of 81.98%, and F1 of 87.52%. The pharmacologic substance HNER performance of BiLSTM-CRF (word + char + POS) has a precision of 94.85%, recall of 73.47%, and F1 of 84.51%. BiLSTM-CRF (word + char) has a precision of 94.93%. Experimental results on the presented dataset show that BiLSTM-CRF (word + char + POS) could yield excellent performance for the HNER task. Surprisingly, the precision of BiLSTM-CRF without the POS tagging model for disease or syndrome is 0.54% higher, and for pharmacologic substance it is 0.08% higher than that of the BiLSTM-CRF with the POS tagging model when evaluating the presented dataset. Also, the F1 of LSTM-CRF with the all-features model for disease or syndrome is 0.31% higher than the BiLSTM-CRF with the-features model.

For these experiments, we used “Pyysalo Wiki + PM + PMC” word embeddings that achieve higher results than other pre-trained word embeddings (see Table 8). As compared to the Micromed dataset and the presented dataset, the LSTM + CRF (word) model applied to both datasets. The model on the presented dataset improved the performance significantly. LSTM+CRF (word) model performed better results than LSTM + CRF (char) and LSTM + CRF (POS) models. We can see that word embedding is most effective feature for HNER task compared with character embedding and POS tagging. The models with different combinations of features improve the result. The best results are shown with BiLSTM-CRF (word + char + POS), using the combination of all feature types. The Twitter dataset is highly noisy and many out-of-vocabulary words are contained. Because of that, character embedding helps to learn more those words and other rare words. As we mentioned above, most of the state-of-the-art results used POS tagging. Also, our experimental result proves that POS tagging is efficient in various NER tasks. Generally, the BiLSTM + CRF model outperforms the LSTM + CRF model in all the experiments.

As shown in Table 7, pre-trained word embedding is the most significant feature and can be used efficiently for down-stream tasks such as NER and HNER tasks. We achieved the best result with BiLSTM-CRF (word + char + POS) model. We studied the contribution of medical and non-medical word embeddings to BiLSTM-CRF (word +char + POS) model performance by removing each of them in turn from the model and then evaluating the model on the presented dataset. In this regard, we evaluate the model with character embedding and POS tagging. Table 8 shows the predictive performance for the model with different word embeddings on the testing set. Generally, the models with non-medical pre-trained word embeddings achieve a higher result than medical pre-trained word embeddings. The experimental results show that medical word embeddings help the model to boost its performance for disease or syndrome, sign or symptom, and pharmacologic substance HNER tasks. We ranked the word embeddings by the performance as follows: (1) “Pyysalo Wiki + PM + PMC” achieved the highest result in 6/9 experiments, (2) “Chen PM + MIMIC III” achieved the highest result in 2/9 experiments, and (3) “Pyysalo PM + PMC” achieved the highest result in 1/9 experiments. Those three word embeddings are even more powerful than the rest of the embeddings together in the disease or syndrome, sign or symptom, and pharmacologic substance HNER with BiLSTM-CRF (word + char + POS) model.

The contribution of word embeddings to recognition of each named entity type is also different. “Chen PM + MIMIC-III” has more effect in recognition of disease or syndrome named entities than of the other named entities. “Pyysalo Wiki + PM + PMC” has more effects in the recognition of sign or symptom and pharmacologic substance named entities than of the other named entity.

We also examined the impact of fine-tuning embeddings in disease or syndrome, sign or symptom, and pharmacologic substance HNER by comparing the performance of BiLSTM-CRF (word + char + POS) model with that of an variant of it, in which “Pyysalo Wiki + PM + PMC” and “Chen PM + MIMIC-III” word embeddings are not fine-tuned during the model training as shown in Table 9. The comparative results of two word embeddings with the model on the presented dataset demonstrate that fine-tuning embeddings has a certain effect on the performance of BiLSTM-CRF (word + char + POS) model. The F1 of BiLSTM-CRF with “Pyysalo Wiki + PM + PMC” is improved for disease or syndrome, sign or symptom, and pharmacologic substance HNER when the model uses fine-tuned embeddings, i.e., 0.99%, 1.45%, and 1.95%, respectively. The F1 of BiLSTM-CRF with “Chen PM + MIMIC III” is improved for disease or syndrome, sign or symptom, and pharmacologic substance HNER when the model uses fine-tuned embeddings, i.e., 0.39%, 1.16%, and 0.92%, respectively.

## 5. Conclusions

In this paper, we discuss advanced neural networks methods known as BiLSTM-CRF that are able to achieve the health-related NER task with word embedding, character embedding, and small feature engineering with POS tagging. The ontology or knowledge base is important for learning about the medical domain. Our goal is to predict and recognize medical terms in tweets that support public health systems. We annotated the collected dataset by using UMLS metathesaurus ontology to obtain knowledge about the specific domain. We considered three entity types: disease or syndrome, sign or symptom, and pharmacologic substance.

In the scope of HNER task, we presented a dataset collected from Twitter using the search term “healthcare” between 12 July 2018 and 12 July 2019, obtaining 676,251 tweets, 1,330,867 medical terms, and 19,727 unique medical terms. The presented dataset is larger than the previously presented dataset known as Micromed. The size of the dataset significantly improves the performance of the models. To produce the experimental dataset, we used the preprocessing techniques on the raw text data (tweets) such text cleaning, normalization, filtering, and removing non-medical terms and tokenization.

Inspired by this kind of work, we employed the BiLSTM-CRF model and compared with LSTM-CRF model with different combinations of features such as word embedding, character embedding, and POS tagging. Bidirectional models learn the input features in two ways: one from the beginning to end, and other from end to beginning, helping the learning of the feature more efficiently. We found that the BiLSTM-CRF (word + char + POS) model achieves the best result compared with other models on the HNER task when using “Pyysalo Wiki + PM + PMC” pre-trained word embeddings. The best model achieves a precision of 93.99%, recall of 73.31%, and F1-score of 81.77% for disease or syndrome HNER; a precision of 90.83%, recall of 81.98%, and F1-score of 87.52% for sign or symptom HNER; and a precision of 94.85%, recall of 73.47%, and F1-score of 84.51% for pharmacologic substance named entities. We also proved that fine-tuning is efficient when working on down-stream NLP tasks such as HNER.

As we found BiLSTM-CRF with “Pyysalo Wiki + PM + PMC” word embeddings, CNN-based character embedding and POS tagging is the best model for prediction of disease or syndrome, sign or symptom, and pharmacologic substance named entities.

In the future, we will extend the HNER task by adding different types of medical entities from UMLS entity types. We will apply transformer networks like BERT, ELMO, XLNET, etc. on the HNER tasks that currently dominate in most NLP tasks.

## Figures and Tables

**Table 1:** Medical entity types.

Type ID	Entity Type	Total Tweets	Total Entities	Unique Entities
T047	Disease or syndrome	189,517	382,629 (7.25%)	9536 (3.74%)
T184	Sign or symptom	77,466	99,367 (4.33%)	2043 (1.56%)
T121	Pharmacologic substance	409,268	848,871 (7.51%)	8148 (1.80%)

**Table 2:** Example of unique terms. Top most frequent terms per entity type.

No	Disease or Syndrome		Sign or Symptom		Pharmacologic Substance	
1	diabetes	42,493	pain	7355	pharmaceutical	42,688
2	malnutrition	6541	amotivation	7036	digitalis	27,882
3	Alzheimer’s	4402	depression	3387	cannabis	17,782
4	obesity	3667	illness	2614	radiopharmaceutical	12,691
5	flu	2593	out toe	2303	therex	9112
6	coinfection	2556	anxiety	2142	marijuana	8979
7	pregnancy	1889	strain	1510	providine	6190
8	devic	1683	in toe	1506	pediatric	5701
9	blight	1549	tired	1120	nonprescription	5571
10	asthma	1517	ill	1007	californium	5385

**Table 3:** Distribution of experimental datasets.

Subset	Type ID	Total Tweets	Total Terms	Unique Terms
Training	T047	125,275	215,326	7766
	T184	47,554	56,105	1665
	T121	287,341	477,408	6559
Validation	T047	53,096	71,686	5141
	T184	17,436	18,660	1061
	T121	125,012	159,099	4073
Testing	T047	67,137	95,558	5797
	T184	22,874	24,846	1195
	T121	158,064	212,257	4685

**Table 4:** Dataset annotation.

Parameters	Value
quickumls_fp	UMLS data files (umls-2019AA-metathesaurus)
overlapping_criteria	“score”
threshold	0.7
similarity_name	“jaccard”
window	5
accepted_semtypes	“T047”, “T184”, “T121”

**Table 5:** Additional POS features. IN: preposition or subordinating conjunction; PRP: presonal pronoun; RB: adverb; VBP: verb, non-third person singular present; JJ: adjective; NN: noun, singular or mass.

Tweet	if	you	ever	feel	unwell	fart	your	way	into	wellness	health
**POS**	IN	PRP	RB	VBP	JJ	RB	PRP	NN	IN	JJ	NN

**Table 6:** The parameters for our experiments.

Hyper-Parameter	Values
Convolution width	3
CNN output size	50
LSTM state size	250
LSTM layers	2
Learning rate	0.001
Epochs	100
Dropout	0.5
Batch size	100

**Table 7:** The predictive performance for different models on the testing set.

Model	Disease or Syndrome			Sign or Symptom			Pharmacologic Substance		
	P	R	F	P	R	F	P	R	F
LSTM-CRF (word)	91.30	68.44	78.29	86.18	76.85	82.32	91.39	68.57	79.63
LSTM-CRF (char)	90.86	67.78	77.63	85.15	75.89	82.11	90.88	67.53	79.12
LSTM-CRF (POS)	90.05	67.15	77.02	84.14	75.61	81.08	90.07	67.07	78.61
LSTM-CRF (word + char)	92.75	70.24	81.60	88.12	78.76	85.03	93.55	71.11	82.06
LSTM-CRF (word + POS)	92.42	68.82	79.44	86.74	77.91	84.06	92.65	70.53	82.01
LSTM-CRF (char + POS)	92.07	68.68	78.43	86.52	77.21	82.88	92.39	69.44	80.48
LSTM-CRF (word + char + POS)	93.85	71.27	**82.08**	88.59	79.44	85.38	93.97	71.37	82.22
BiLSTM-CRF (word)	93.01	69.60	79.35	87.29	77.86	83.52	93.08	70.07	81.31
BiLSTM-CRF (char)	92.08	69.07	79.21	86.58	76.90	83.14	92.06	69.10	80.42
BiLSTM-CRF (POS)	91.69	68.71	78.26	85.35	76.64	82.39	91.51	68.26	79.99
BiLSTM-CRF (word + char)	**94.53**	72.52	81.72	89.15	80.21	86.22	**94.93**	72.27	83.06
BiLSTM-CRF (word + POS)	93.54	70.51	81.07	89.00	79.39	85.22	94.13	71.06	83.05
BiLSTM-CRF (char + POS)	93.24	69.69	79.89	87.72	78.33	84.15	93.42	68.87	82.45
BiLSTM-CRF (word + char + POS)	93.99	**73.31**	81.77	**90.83**	**81.98**	**87.52**	94.85	**73.47**	**84.51**

Note: The best results are highlighted in bold. word: word embedding; char: character embedding.

**Table 8:** Impact of different word embeddings of BiLSTM-CRF (word + char + POS) model. Wiki: Wikipedia; GW: gigaword; CC: common crawl; PM: pubmed; PMC: pubmed central; win: windows; dim: dimension; MIMIC: Medical Information Mart for Intensive Care.

Word Embedding	Disease or Syndrome			Sign or Symptom			Pharmacologic Substance		
	P	R	F	P	R	F	P	R	F
Glove Wiki + GW [55]	89.87	68.11	77.10	86.72	77.06	82.87	90.11	68.93	80.32
Glove CC-42 [55]	89.35	67.42	76.77	85.64	76.63	82.40	89.80	68.41	79.59
Glove CC-840 [55]	89.64	67.71	76.76	86.57	76.66	82.58	89.90	68.85	80.27
Glove Twitter [55]	90.94	69.40	78.33	88.09	78.55	83.98	91.13	70.31	81.41
Word2Vec [56]	89.16	67.38	76.40	85.07	76.46	82.22	89.65	67.67	78.68
Pyysalo PM [57]	91.00	69.60	79.23	88.38	79.74	85.45	91.23	70.33	82.80
Pyysalo PMC [57]	91.71	69.64	80.19	88.76	80.21	85.93	91.33	70.86	83.04
Pyysalo PM + PMC [57]	93.55	72.19	80.89	89.95	80.55	86.04	92.89	71.00	**84.81**
Pyysalo Wiki + PM + PMC [57]	93.99	**73.31**	81.77	**90.83**	**81.98**	**87.52**	**94.85**	**73.47**	84.51
Chiu win-2 [47]	92.94	70.58	80.59	89.62	81.04	86.22	92.33	71.74	82.75
Chiu win-30 [47]	93.26	70.67	80.77	89.81	81.15	86.65	92.53	72.17	82.89
Chen PM + MIMIC III [58]	**94.68**	71.88	**82.13**	90.47	81.17	84.51	92.97	73.10	83.37
Aueb dim-200 [59]	91.79	70.10	78.65	88.09	80.69	84.25	94.40	72.76	82.29
Aueb dim-400 [59]	92.56	70.51	78.91	88.41	80.80	84.41	94.67	73.01	82.68

Note: The best results are highlighted in bold.

**Table 9:** Impact of fine-tuning embeddings of BiLSTM-CRF (word + char + POS) model.

Word Embedding	Disease or Syndrome			Sign or Symptom			Pharmacologic Substance		
	P	R	F	P	R	F	P	R	F
Pyysalo Wiki + PM + PMC									
Not fine-tuned	92.24	71.84	80.78	88.87	79.87	86.07	93.08	71.41	82.56
Fine-tuned	93.99	73.31	81.77	90.83	81.98	87.52	94.85	73.47	84.51
Chen PM + MIMIC III									
Not fine-tuned	91.37	70.21	81.74	88.45	80.87	83.35	92.81	72.68	82.45
Fine-tuned	94.68	71.88	82.13	90.47	81.17	84.51	92.97	73.10	83.37

## Appendix A. Word Embedding

Word embedding is mainly learned through context and the learned word vectors can capture general syntactic and semantic information. Those word vectors have proven to be efficient in capturing context, semantic similarity, and analogies; due to their smaller dimensionality, they are fast and efficient in text mining tasks [55,66]. Typically, word embedding is pre-trained by optimizing an auxiliary objective in a large unlabeled corpus which is used for other downstream tasks. Following the popularization of word embedding and its ability to represent the semantic relationship between entities in a distributed space, an effective feature learning function is needed to extract higher-level features from the word embedding. The statistics of word embedding are described in Table A1 and Table A2.

**Table A1:** Non-biomedical word embedding.

Word Embedding	Vocabulary	Dimension
Glove Wiki + GW [55]	400K	300
Glove CC-42 [55]	1.9M	300
Glove CC-840 [55]	2.2M	300
Glove Twitter [55]	1.2M	200
Word2Vec [56]	3M	300

**Table A2:** Biomedical word embedding.

Biomedical		
Pyysalo PM [57]	2.3M	200
Pyysalo PMC [57]	2.5M	200
Pyysalo PM + PMC [57]	4M	200
Pyysalo Wiki + PM + PMC [57]	5.4M	200
Chiu win-2 [47]	2.2M	200
Chiu win-30 [47]	2.2M	200
Chen PM + MIMIC III [58]	16.5M	200
Aueb dim-200 [59]	2.6M	200
Aueb dim-400 [59]	2.6M	400

## Appendix B. Character Embedding

We randomly initialized a lookup table with values drawn from a uniform distribution with range (−0.5, 0.5) to output character embedding of 25 dimensions. The character set includes numbers, upper and lower case English alphabets, some special characters, and the special tokens padding (PAD) and unknown (UNK), as shown in Table A3. The PAD token is used for the CNN, and the UNK token is used for all other characters. The CNN extracts a fixed length of feature vector from character-level features. The character embedding is computed through lookup tables.

**Table A3:** Character set.

Numbers	0 1 2 3 4 5 6 7 8 9	10
Lower alphabets	a b c d e f g h i j k l m n o p q r s t u v w x y z	26
Uppercase alphabets	A B C D E F G H I J K L M N O P Q R S T U V W X Y Z	26
Punctuation	. , - _ ( ) [ ] { } ! ? : ; # ‘ \ “ / \ \ % $ ` & = * + @ ^ ~ |	32

Then, they are concatenated and passed into CNN. The architecture of character-level feature extraction using CNN is shown in Figure A1.

The CNN is similar to the one in Chiu et al. [50], except that we use only character embedding as the input to CNN, without any character type features. For each word, we employ a convolution and a max-pooling layer to extract a new feature vector from the character embedding. Words are padded with a number of special PAD characters on both sides depending on the window size of the CNN. The hyper-parameters of the CNN are the window size and the output vector size.

The advantage of the character-based approaches is their language and domain independence, since they do not require any language and domain specific parsing. With character embedding, every single word’s vector can be formed even it is out of the vocabulary (optional). On the other hand, word embedding can only handle those seen words.

## Appendix C. BiLSTM

Recurrent neural networks (RNNs) [67] are a family of neural networks. RNNs have a high-dimensional hidden state with non-linear dynamics that encourage them to take advantage of previous information. Gated RNNs are the most effective sequence models in practical applications, including LSTM [68]. LSTM can address the vanishing and exploding gradient problems by adding extra memory cell inherent in RNNs. LSTM networks are the same as RNNs, except that the hidden layer updates are replaced by purpose-built memory cells. As a result, they may be better at finding and exploding long-range dependencies in the data.

Given a sentence, the model predicts a label corresponding to each of the input tokens in the sentence. Firstly, through the embeddings layer, the sentence is represented as a sequence of vectors $X=\left(x_{1},\;\ldots ,\;x_{t},\;\ldots ,\;x_{n}\right)$ where n is the length of the sentence. Next, the embeddings are given as input to a BiLSTM [69] layer which is composed of LSTM memory cell. Figure A2 illustrates a single LSTM memory cell. The LSTM memory cell is implemented as the following:

(A1) $$i_{t}=\sigma \left(W_{xi}x_{t}+W_{hi}h_{t-1}+W_{ci}c_{t-1}+b_{i}\right)$$

(A2) $$f_{t}=\sigma \left(W_{xf}x_{t}+W_{hf}h_{t-1}+W_{cf}c_{t-1}+b_{f}\right)$$

(A3) $$c_{t}=f_{t}c_{t-1}+i_{t}\tan h\left(W_{xc}x_{t}+W_{hc}x_{t-1}+b_{c}\right)$$

(A4) $$o_{t}=\sigma \left(W_{xo}x_{t}+W_{ho}h_{t-1}+W_{co}c_{t}+b_{o}\right)$$

(A5) $$h_{t}=o_{t}tanh\left(c_{t}\right)$$

where W is weight matrix, b is bias, σ is the logistic sigmoid function, and i, f, c, o are the input gate, forget gate, cell vectors, and output gate, respectively, all of which are the same size as the hidden vector h.

In the BiLSTM layer, a forward $\vec{LSTM}$ computes a representation $\vec{h_{t}}$ of the sequence from left to right at every word t, and another backward $\overset{\leftarrow }{LSTM}$ computes a representation $\overset{\leftarrow }{h_{t}}$ of the same sequence in reverse.

(A6) $$\vec{h_{t}}=\vec{LSTM}\left(x_{t},\;\vec{h_{t-1}}\right),t\in \left[1,\;n\right]$$

(A7) $$\overset{\leftarrow }{h_{t}}=\overset{\leftarrow }{LSTM}\left(x_{t},\;\overset{\leftarrow }{h_{t+1}}\right),\;t\in \left[1,\;n\right]$$

These two distinct networks use different parameters, and then the representation of a word $h_{t}{}^{concat}=\left[\vec{h_{t}};\overset{\leftarrow }{h_{t}}\right]$ is obtained by concatenating its left and right context representations. Then a tanh layer on top of the BiLSTM is used to predict confidence scores (CS) for the word with each of the possible labels as the output score of the network.

(A8) $$CS_{t}=tanh\left(W_{e}h_{t}{}^{concat}\right)$$

(A9) $$CS_{t}=tanh\left(W_{e}h_{t}{}^{concat}\right)$$

where the weight matrix $W_{e}$ is the parameter of the model to be learned in training.

## Appendix D. CRF

Finally, instead of tagging decisions independently, the CRF [70] layer is added to decode the best tag path in all possible tag paths. We consider S to be the matrix of scores output by the network. The $i^{th}$ column is the vector $CS_{t}$ obtained by Equation (A5). The element $S_{i,j}$ of the matrix is the score of the $j^{th}$ tag of the $i^{th}$ word in the sentence. We used a tagging transition matrix T, where $T_{i,j}$ represents the score of transition from tag i to tag j in successive words and $T_{0,j}$ as the initial score for starting from tag j. This transition matrix will be trained as the parameter of the model. The score of the sentence X along with a sequence of predictions $y=\left(y_{1},\;\ldots ,\;y,\;\ldots ,\;y\right)$ is then given by the sum of transition scores and network scores:

(A10) $$s\left(X,\;y\right)=\overset{n}{\underset{i=1}{\sum }}\left(S_{y_{i-1},y_{i}}+S_{i,y_{i}}\right)$$

Then, a softmax function is used to yield the conditional probability of the path y by normalizing the above score over all possible tag paths $\tilde{y}$:

(A11) $$p\left(y|X\right)=\frac{e^{s\left(X,\;y\right)}}{\sum {\tilde{y}}^{s\left(X,\;y\right)}}$$

## Appendix E. Viterbi algorithm

During the training phase, the objective of the model is to maximize the log-probability of the correct tag sequence. At the inference time, we predict the best tag path that obtains the maximum score given by:

(A12) $$\underset{\tilde{y}}{\arg max}s\left(X,\;y\right)$$

This can be computed using dynamic programming, and the Viterbi algorithm [71] is chosen for this inference.
